# Editorial: Technologies to support the diagnosis and therapy of individuals with autism

**DOI:** 10.3389/fpsyt.2023.1304178

**Published:** 2023-11-01

**Authors:** Ahmad Yaser Alhaddad, Wing-Chee So, John-John Cabibihan, Andrea Bonarini

**Affiliations:** ^1^Department of Mechanical and Industrial Engineering, College of Engineering, Qatar University, Doha, Qatar; ^2^Department of Educational Psychology, The Chinese University of Hong Kong Shatin, New Territories, Hong Kong; ^3^Department of Electronics, Information and Bioengineering, Polytechnic University of Milan, Milan, Italy

**Keywords:** autism (ASC), technology—assistive/supportive, robot therapy, social robot, autism therapy, autism diagnosis

The contributions in this Research Topic focus on the recent advances in autism research by exploring recent trends and findings, especially in diagnosis, monitoring, intervention, analysis, therapy, interaction, and training. Most of the studies presented in this Research Topic examine and highlight the importance of incorporating technologies, such as robots, and techniques to support the diagnosis and therapy of individuals with autism.

The first article in this Research Topic, titled “*Digitally assisted diagnostics of autism spectrum disorder*,” and authored by Koehler and Falter-Wagner, explores the potential of digital technologies in improving the diagnostic of autism by providing objective and evidence-based decisions. While the article highlights the benefits of using artificial intelligence combined with automated techniques to identify cognitive, behavioral, and neuronal symptoms, however, it stresses that digital technologies should not replace, but instead complement clinical decision-making by accounting for the complexity and uniqueness of individuals with autism spectrum.

The second article, titled “*Randomized controlled pilot study of an app-based intervention for improving social skills, face perception, and eye gaze among youth with autism spectrum disorder*,” and authored by Chung and Chung, presents and examines the potential of using an app-based intervention aimed to improve social skills, eye gaze, and facial perception in children with autism. The outcomes of this work highlight the effectiveness of the proposed app-based intervention as demonstrated by the improvements in social skills, face perception, and eye gaze.

The third article by Li et al., titled “*Integrated analysis of endoplasmic reticulum stress regulators' expression identifies distinct subtypes of autism spectrum disorder*,” investigates the potential and patterns of endoplasmic reticulum stress regulator in autism. Based on machine learning techniques, the study was able to identify and classify individuals with autism based on their expression profiles. The analysis conducted in this study revealed insights about the stress regulators and highlighted their role in individuals with autism.

The fourth article, titled “*Comparing the effectiveness of robot-based to human-based intervention in improving joint attention in autistic children*,” by So et al., compares the learning effectiveness of robot-based intervention in improving joint attention in children with autism as compared to human-based intervention. The outcomes reveal the potential of considering robot-based intervention in supporting joint attention and social communication skills.

The fifth article “*Global trends and hotspots in the digital therapeutics of autism spectrum disorders: a bibliometric analysis from 2002 to 2022*,” authored by Wu et al., examines the contemporary global digital therapeutics status pertaining to autism research. The article highlights trends incorporating cutting-edge technologies and techniques, such as virtual reality and machine learning, in the development of digital therapeutics for individuals with autism.

The sixth article by Takata et al., and titled “*Social skills training using multiple humanoid robots for individuals with autism spectrum conditions*,” investigates the effectiveness of using multiple humanoid robots to develop a training program to target social skills in individuals with autism. The findings of this work showed the potential and effectiveness of using humanoid robots to support social skills in autism therapy for individual with autism.

The final article, titled “*Development and adaptation of a strength-based job interview training tool for transition age youth on the autism spectrum using community engaged methods*,” and authored by Genova et al., evaluates the feasibility of a job interview training tool aimed to assist adults with autism with interviews. Based on the strength approach, the findings highlight the acceptability, accessibility, and usability of this tool to support showing their strengths and skills in job interviews.

We hope this Research Topic will provide the reader with insightful findings and trends about the recent technological and tools advances in autism research.

## Author contributions

AA: Writing – original draft, Writing – review & editing. W-CS: Writing – original draft, Writing – review & editing. J-JC: Writing – original draft, Writing – review & editing. AB: Writing – original draft, Writing – review & editing.

